# Impaired Function of CD4+ T Follicular Helper (Tfh) Cells Associated with Hepatocellular Carcinoma Progression

**DOI:** 10.1371/journal.pone.0117458

**Published:** 2015-02-17

**Authors:** Yiqiong Jia, Zhen Zeng, Yuanyuan Li, Zhiwei Li, Lei Jin, Zheng Zhang, Lifeng Wang, Fu-Sheng Wang

**Affiliations:** 1 Research Center for Biological Therapy, 302 Military Hospital of China-Peking University Teaching Hospital, Beijing, 100039, P. R. China; 2 Research Center for Biological Therapy, Beijing 302 Hospital, Beijing, 100039, P. R. China; Ohio State University, UNITED STATES

## Abstract

**Background and Aims:**

CD4+ T follicular helper (Tfh) cells, a new subset of immune cells, have been demonstrated to be involved in the development and prognosis of tumors. However, their functional role in human hepatocellular carcinoma (HCC) is relatively unknown, and the detailed mechanisms in HCC development remain to be described.

**Methods:**

A total of 85 HCC patients with hepatitis B virus (HBV) infection, 25 HBV-relative liver cirrhosis (LC) patients, and 20 healthy controls (HC) were randomly enrolled. Flow cytometric analysis, immunohistochemical staining, and relative function (i.e., cytokine secretion, B cell maturation) assays were used to analyze the properties of CXCR5+CD4+ T cells. In addition, the relationship between the frequency of CXCR5+CD4+ T cells and overall survival rates or disease-free survival rates was also analyzed by the Kaplan-Meier method.

**Results:**

The frequency of circulating CXCR5+CD4+ T cells was significantly decreased in HCC patients compared with HBV-relative liver cirrhosis (LC) patients and healthy controls, and the decrease in circulating CXCR5+CD4+ T cells correlated with disease progression. The proportion of infiltrated CXCR5+CD4+ T cells was significantly decreased in tumor regions compared with nontumor regions. Furthermore, compared with healthy controls, the function of circulating CXCR5+CD4+ T cells in HCC was impaired, with reduced IL-21 secretion and dysfunction in promoting B cell maturation. Importantly, follow-up data indicated that a decreased frequency of circulating CXCR5+CD4+ T cells was also associated with reduced disease-free survival time in HCC patients.

**Conclusions:**

Impairment of CD4+ T follicular helper cells may influence the development of HBV-associated HCC. Decreased CD4+ T follicular helper cells may represent a potential prognostic marker and serve as a novel therapeutic target for HCC patients.

## Introduction

Hepatocellular carcinoma (HCC), one of the most common malignancies worldwide, is the third-leading cause of cancer-related deaths [1]. HCC accounts for approximately 70%–80% of all primary liver cancer cases [2] and is characterized by a progressive development and poor prognosis. Recurrence is quite common, and the survival rate is 30%–40% at five years post-surgery [3]. Recent studies have provided evidence that immune system dysregulation plays an important role in the development of HCC [4,5]. Tumor-related immune cells, such as cytotoxic T cells, CD4+ T cells, Treg cells, myeloid-derived suppressor cells (MDSC), and natural killer (NK) cells, have all been reported to be involved in the development of HCC. However, only a few studies have focused on humoral-related immunity [6] in HCC and the regulatory mechanisms.

Th2 cells have been regarded as a key players in orchestrating humoral-related immune responses. Recent studies have demonstrated that an additional effector subset of T follicular helper (Tfh) cells, which are important to B cells during germinal center (GC) reactions in secondary lymphoid tissues [7,8], function to support activation, affinity maturation, and isotype switching, leading to the generation of memory B cells and long-lived plasma cells [9–11]. The distinguishing features of these cells are their high expression of CXCR5, PD-1, ICOS, BCL-6, and CD40L and the cytokine IL-21 and their low expression of CCR7 and IL-7Rα. Human Tfh cells have been implicated in various diseases. Indeed, many reports have shown that the dysregulated behavior of Tfh cells contributes to autoimmune disease, primary immunodeficiency, and acquired immunodeficiency. Recent studies have reported that Tfh cells may have a deep impact on the pathogenesis of various cancers, such as peripheral T-cell lymphoma (PTCL) [12], chronic lymphocytic leukemia [13,14], breast cancer [15], colorectal cancer [16], and nonsmall cell lung cancer [17]. However, little information is available for the association between Tfh cells and HCC or their correlation to HCC progression and survival rates. Furthermore, the regulatory mechanisms responsible for the alterations in Tfh cells in HCC patients also need to be clarified.

To address these issues, 85 HCC patients at different stages of disease progression and with a homogeneous background of HBV-relative liver cirrhosis (LC) were enrolled in this study. The frequency, phenotype, and function of CXCR5+CD4+ Tfh cells in these HCC patients were analyzed. We found that a decreased proportion of CXCR5+CD4+ Tfh cells was associated with HCC disease progression. More importantly, the reduced incidence of CXCR5+CD4+ Tfh cells may represent a promising independent predictor for recurrence in HCC patients.

## Materials and Methods

### Study subjects

Blood samples were obtained from 85 HBV-related HCC patients, age- and sex-matched 25 HBV-related liver cirrhosis (LC) patients, and 20 healthy donors. All the HCC patients had a history of more than 10 years of chronic HBV infection and were hospitalized or followed up at Beijing 302 Hospital. The diagnosis of HCC was based on the results of standard biopsies or imaging according to the American Association for the Study of Liver Diseases (AASLD) guidelines [18]. A diagnosis of tumor recurrence after resection was based on imaging. Tumor-infiltrating lymphocytes (TILs) and nontumor-infiltrating lymphocytes (NILs) were isolated from the liver tissues of 13 HCC patients who had undergone surgical resection or liver transplantation. None of the patients received anticancer therapy prior to the sampling. The clinical background of the HCC patients was shown in Table 1. The criteria for the staging of primary HCC was according to Barcelona Clinic Liver Cancer (BCLC) [19]. The study protocol was approved by Beijing 302 Hospital Research Ethics Committee, and written informed consent was obtained from each subject prior to blood and tumor sampling. Concurrence of hepatitis C virus, hepatitis D virus, human immunodeficiency virus infection, and autoimmune or alcoholic liver disease were exclusion criteria for all enrolled individuals.

**Table 1:** Clinical characteristics of the enrolled 85 of HBV-related HCC patients.

Variable	Results
Age (years)	51 (35–66)
Gender (male/female)	73/12
HBsAg (+/-)	85/0
HBsAb (+/-)	0/85
HBeAg (+/-)	29/56
HBeAb (+/-)	52/33
HBcAb (+/-)	85/0
HBV DNA (+/-/ND)	29/49/7
Child-Pugh classification (A/B/C)	59/23/3
AFP (>400/400–200/<200)	35/7/43
BCLC (0/A/B/C/D)	2/18/23/40/2

HBsAg, hepatitis B surface antigen; HBsAb, hepatitis B surface antibody; HBeAg, hepatitis B e antigen; HBeAb, hepatitis B e antibody; HBcAb, hepatitis B core antibody; ND, no data; AFP, α-fetoprotein; BCLC, Barcelona Clinic Liver Cancer.

### Cell isolation

Peripheral blood mononuclear cells (PBMCs) were isolated from freshly obtained heparinized blood by Ficoll–Hypaque density gradient centrifugation. The total cell number was counted in the presence of trypan blue dye to evaluate cell viability. HCC tumor tissues and adjacent non-tumor tissues (at least 1 cm distant from the tumor) were obtained from surgical specimens immediately after resection from patients undergoing primary surgical treatment of HCC in Beijing 302 Hospital. Those tissues were carefully washed with Hank’s solution containing 2% FBS and 1% EDTA to remove peripheral blood, cut into small pieces, homogenized, and placed between two semi-frosted microscopic slides. The dissociated cell suspension was mixed and kept on ice for 15 min (at the bottom of tube, the pellet was mainly composed of liver tissue debris, while in the suspension, lots of lymphocytes were remain in there). The upper part of the suspension was carefully recovered, passed through a 70-μm cell strainer (BD Labware), and laid on Ficoll-Hypaque separation solution. Then, following the slandered protocol, tumor-infiltrating lymphocytes (TILs) and nontumor-infiltrating lymphocytes (NILs) were isolated by density gradient centrifugation at last. The viability of the isolated cells was determined by trypan blue exclusion staining. In general, ˃ 1×10^6^ liver-infiltrating lymphocytes (LILs) could be obtained from 1 g of liver tissue, and the viable TILs and NILs were ˃ 95%.

### Flow cytometric analysis

Fluorescein isothiocyanate (FITC), phycoerythrin (PE), peridinin chlorophyll protein (PerCP), and allophycocyanin (APC) are different fluoresceins. PE-conjugated anti-ICOS, Alexa Fluor 488-conjugated, and Alexa Fluor 647-conjugated anti-CXCR5 were purchased from BD PharMingen (San Diego, CA, USA). APC-conjugated anti-PD-1, PE/Cy7-conjugated anti-CD45, PE-conjugated anti-IL-21, PE/Cy7-conjugated anti-CD19, APC/cy7-conjugated anti-CD3, APC/Cy7-conjugated anti-CD27, and PerCP/Cy5.5-conjugated anti-IgD were purchased from Biolegend (San Diego, CA, USA). eFluor 660-conjugated anti-IL-10 and APC-conjugated anti-IL-17A were purchased from eBioscience (San Diego, CA, USA). PerCP-conjugated anti-CD4 and anti-CD8 were purchased from BD Biosciences (San Diego, CA, USA).

To determine the frequency of Tfh cells and the surface marker profile, PBMCs (at least 1×10^6^ cells/tube) were stained with mAbs for 30 min on ice. Appropriate isotype antibody controls were used for each sample. The cells were washed and examined by four-color flow cytometry. For intracellular cytokine staining, 300 μl blood and 700 μl RPMI1640/tube were stimulated with phorbol 12-myristate 13-acetate (PMA) (300 ng/ml) and ionomycin (1 μg/ml) and then cultured at 37°C in a humidified CO_2_ incubator for 6 h. The activated cells were first incubated with anti-CD8-PerCP, anti-CD3-APC/Cy7, and anti-CXCR5-Alexa Fluor 488 (or anti-CXCR5-Alexa Fluor 647) for 20 min at room temperature, followed by fixation and permeabilization of the activated cells for 50 min at 4°C with eBioscience Fixation/Permeabilization Buffer (eBioscience, San Diego, CA, USA). The samples were then stained with anti-IFN-γ-FITC, anti-IL-21-PE, anti-IL-17A-APC, and anti-IL-10-APC for 20 min each at room temperature. Appropriate isotype antibody controls were included for each sample. After staining, the cells were fixed in 1% paraformaldehyde, and eight-color flow cytometric analyses were performed using FACSverse and CELLQuest software (Becton Dickinson, San Jose, CA, USA).

### Immunohistochemical staining of Tfh cells in liver tissue

Paraffin-embedded, formalin-fixed liver tissue was cut into 5-μm sections and placed on polylysine-coated slides. Antigen (Ag) retrieval was achieved by pressure cooking for 10 min in citrate buffer (pH 6.0). Anti-human CD3 (Zhongshan Goldenbridge Biotech, Beijing, China) or rabbit–anti-human CXCR5 (Santa Cruz Biotechnology, San Jose, CA, USA) polyclonal antibodies were used for CD3 and CXCR5 staining, respectively, followed by biotinylated goat anti-rabbit Ig (Zhongshan Goldenbridge Biotech, Beijing, China). 3-Amino-9-ethyl-carbazole (red color) was used as the substrate, followed by counterstaining with hematoxylin for single staining. Double staining was performed using the avidin-biotin-peroxidase system with 2 different substrates: Vector blue (blue color) for CD3 and 3-amino-9-ethyl-carbazole for CXCR5. The Tfh cells were evaluated quantitatively by analyzing at least 3 different fields (200×) by 2 independent observers.

### Co-culture of Tfh cells and B cells

Circulating CXCR5+CD4+ Tfh cells from 10 HCC patients (pTfh) or 10 healthy controls (hTfh) and allogeneic CD19+ B cells from 10 healthy controls (hB) were sorted using a FACSAria II cell sorter and incubated with each other at a ratio of 1:1 in the present of SEB (100 ng/ml) in RPMI1640 complete medium supplemented with 2 mM glutamine, 1%(v) nonessential amino acids, 1% sodium pyruvate, penicillin (50 U/ml), streptomycin (50 μg/ml), kanamycin (50 μg/ml), and 10%(v) fetal bovine serum (all from Hyclone) [20,21]. After 7–8 days of culture, the B cell subpopulations were examined based on surface phenotypes.

### Statistical analysis

The data analysis was performed with SPSS version 13.0 for Windows software (Chicago, IL, USA); the mean ± standard deviation (SD) for percentages was provided. The statistical significance of difference between the 2 groups was determined by applying the Mann-Whitney nonparametric U test. Multiple comparisons were made between the different groups with Kruskal-Wallis H nonparametric test. Correlation analysis was evaluated by the Spearman rank correlation test. Actuarial overall survival (OS) rates were analyzed by the Kaplan-Meier method, and survival was measured in weeks from diagnosis to death or the last review for the patients who did not receive any antitumor therapy from diagnosis to death. Disease-free survival (DFS) was measured in weeks from resection to tumor recurrence or the last observation. The log-rank test was applied for comparisons between groups. In all tests, *p* < 0.05 was considered to be significant.

## Results

### CXCR5+CD4+ Tfh are significantly decreased in HCC patients

**Fig 1:**
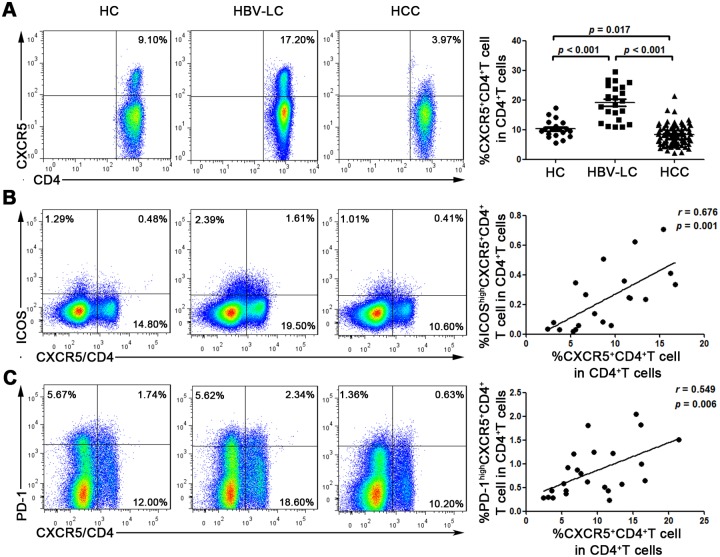
Circulating CXCR5+CD4+ Tfh cells are significantly decreased in HCC patients. (A) Representative prevalence of CXCR5+CD4+ Tfh cells from individual subjects in 3 studied groups and statistical analysis showing that the frequency of CXCR5+CD4+ Tfh cells in HCC patients is significantly lower than in HBV-LC patients and healthy controls. Each dot represents one individual. (B and C) Representative prevalence of ICOS^high^CXCR5+CD4+ and PD-1^high^CXCR5+CD4+ Tfh cells from individual subjects in 3 studied groups, significant correlations are found between the frequency of CXCR5+CD4+ Tfh cells and ICOS^high^, PD-1^high^. Each dot represents one individual. The solid line represents the linear growth trend. r, correlative coefficient. *p* values are shown.

Using flow cytometry, we detected the frequency of circulating CXCR5+CD4+ Tfh cells in 85 HCC, 25 HBV-LC patients, and 20 healthy controls. As shown in Fig. 1A, a significantly decreased frequency of circulating Tfh cells was found in HCC patients compared with the HBV-LC patients (mean, 8.4% ± 3.4% vs 19.3% ± 5.8%, p ˂ 0.001) and healthy controls (vs 10.4% ± 3.0%, p = 0.017). In addition, we also investigated two other important surface markers in Tfh cells (ICOS, PD-1) and found that the proportion of ICOS^high^ (r = 0.676, p = 0.001) and PD-1^high^ (r = 0.549, p = 0.006) showed a positive correlation with the percentage of Tfh cells (Fig. 1B and 1C). Meanwhile, we also tested other markers (such as CD40L, BTLA, CD38, CD69, CD25, HLA-DR) of Tfh cells and their relative subgroups (naïve and memory). No significant differences were found between HCC patients and healthy controls for most of them, except for CD38 (S1 Fig.).

**Fig 2:**
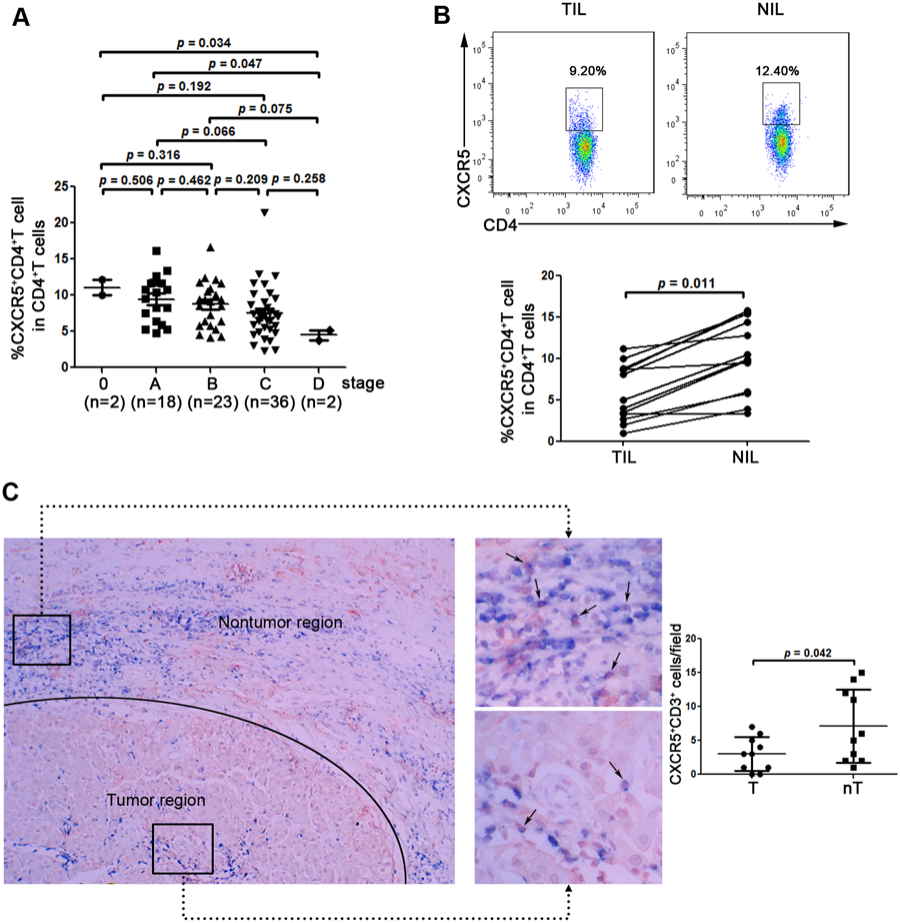
A decrease in circulating CXCR5+CD4+ Tfh cells is associated with HCC progression, and CXCR5+CD4+ Tfh cells are decreased in tumor regions. (A) The prevalence of CXCR5+CD4+ Tfh cells decreases with progressive stages in HCC. Each dot represents one individual. *p* values are shown. (B) Comparison of TILs with NILs shows that the frequency of CXCR5+CD4+ Tfh cells decreased significantly in the former. (C) Representative immunohistochemical staining of samples from HCC patients. Tfh cells are double stained for CD3 (blue, on cell membrane) and CXCR5 (red, on cell membrane). Quantitative analysis of Tfh-positive dots in tumor region and nontumor region from HCC patients. The data are collected at 200× power by two different investigators. One dot indicates one section from one patients. T, tumor region; nT, nontumor region.

Most importantly, we found that the frequency of Tfh cells was significantly decreased in advanced stages (Stage D) compared with early stages (stage 0, 4.5% ± 0.9% vs 11.1% ± 1.5%, *p* = 0.034; or stage A, 4.5% ± 0.9% vs 9.5% ± 3.2%, *p* = 0.047) (Fig. 2A) of HCC. These results might indicate that a decreased frequency of circulating Tfh cells correlated with HCC progression.

Furthermore, the proportions of TILs and NILs of infiltrated Tfh cells in the HCC patients were also investigated. As shown in Fig. 2B, a higher percentage of infiltrated Tfh cells was found to be NILs, as opposed to TILs (10.2% ± 4.4% vs 5.9% ± 3.4%, *p* = 0.011) in these patients. To visualize Tfh cells in the tumor site, immunohistochemical staining was also performed, and the cells that were double positive for CD3 and CXCR5 were defined as Tfh cells in liver tissues. Consistent with the flow cytometry data, liver Tfh cells preferentially accumulated in nontumor regions compared with tumor regions (Fig. 2C). These data indicated that Tfh cells in HCC liver tissues may be involved in the process of HCC development, the underlying mechanism of which requires further study.

### Circulating CXCR5+CD4+ Tfh frequency is associated with AFP levels in HCC patients

**Fig 3:**
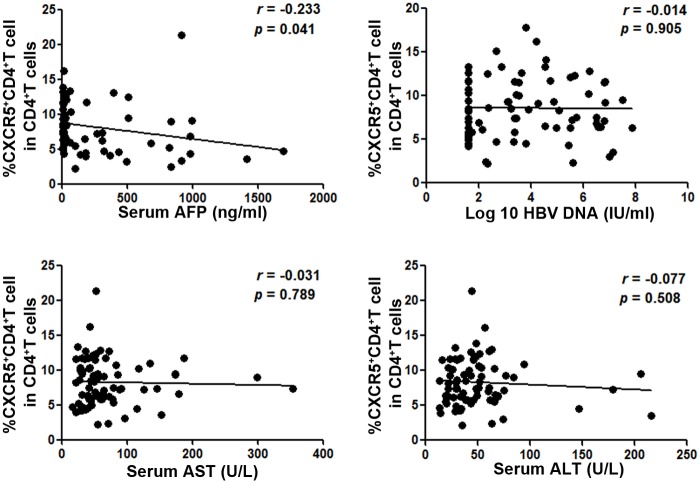
Correlations between the frequency of CXCR5+CD4+ Tfh cells and clinical parameters. The solid line represents the linear growth trend. r, correlative coefficient. *p* values are shown. AFP, α-fetoprotein; AST, aspartate aminotransferase; ALT, alanine aminotransferase.

The plasma HBV DNA load and serum α-fetoprotein (AFP) level are both reported to be related to the development of HCC. In addition, liver biochemical parameters (e.g., serum alanine aminotransferase (ALT) levels and serum aspartate aminotransferase (AST) levels) are elevated in the inflammation state and also related to tumorigenesis. Thus, we analyzed the correlation between the frequency of Tfh cells and all the above-mentioned parameters in the HCC patients. There was a significant negative correlation between the frequency of Tfh cells and serum AFP levels (r = -0.233, *p* = 0.041), but no correlations were found with the plasma HBV DNA load (r = -0.014, *p* = 0.905), serum AST levels (r = -0.031, *p* = 0.789), and serum ALT levels (r = -0.077, *p* = 0.508) (Fig. 3).

### The function of circulating CXCR5+CD4+ Tfh is impaired in HCC patients

**Fig 4:**
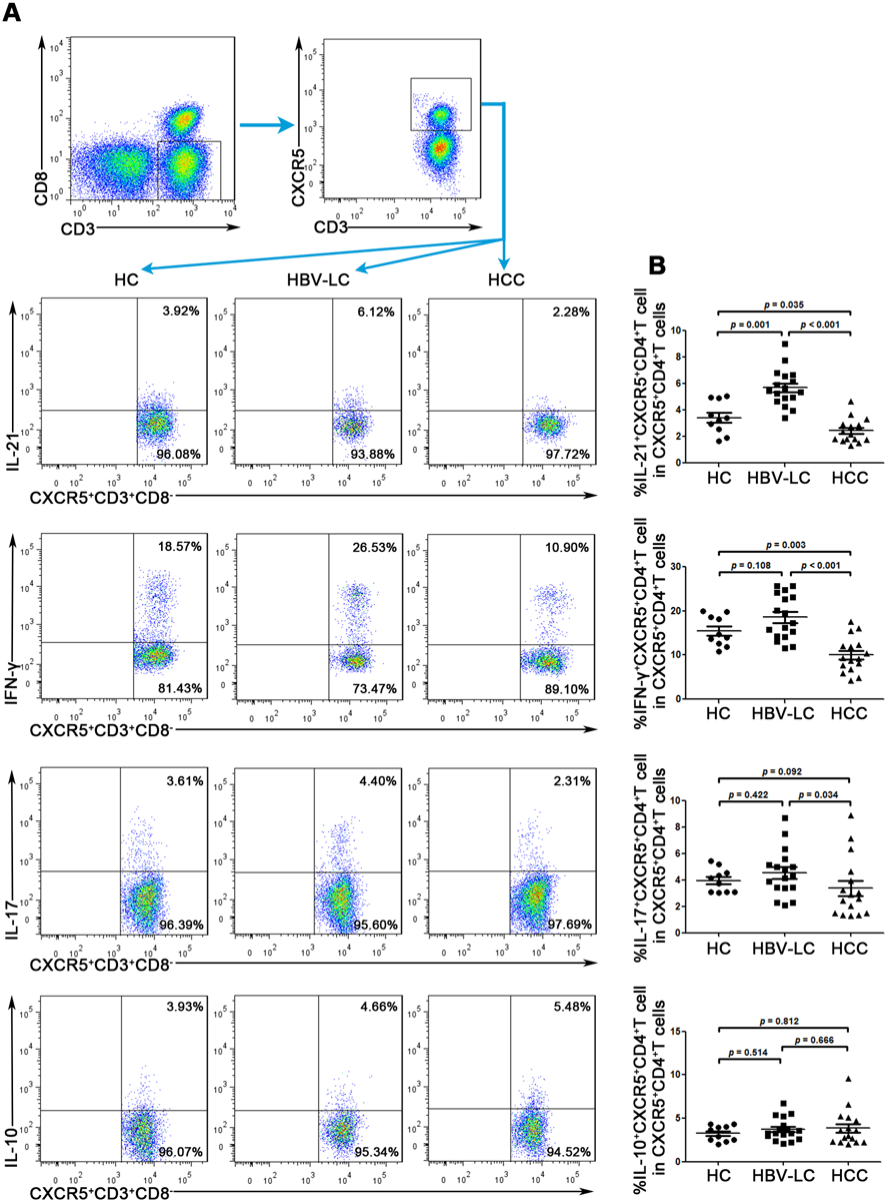
Tfh cells from HCC patients secrete significantly lower levels of IL-21 and IFN-γ than Tfh cells from HBV-LC individuals and healthy controls. (A) Representative dot plots depict IL-21, IFN-γ, IL-17, and IL-10 expression on peripheral cells among the 3 groups in response to PMA/Iono stimulation. (B) Pooled data showing that the percentages of IL-21 and IFN-γ secreted by Tfh cells in HCC patients are significantly lower than in HBV-LC patients and healthy controls. There are no significant differences regarding IL-17 and IL-10 secreted by Tfh cells between the HCC and HBV-LC patients and healthy controls. Each dot represents one individual. *p* values are shown.

As previously reported, cytokines, especially IL-21, play a very important role in Tfh cell functions [22,23]. In the present study, we found that the IL-21 levels secreted by Tfh cells from HCC patients were significantly decreased compared with those from HBV-LC individuals (2.4% ± 0.9% vs 5.7% ± 1.4%, *p* ˂ 0.001) and healthy controls (2.4% ± 0.9% vs 3.4% ± 1.2%, *p* = 0.035) (Fig. 4). In contrast, the IL-21 levels secreted by Tfh cells from HBV-LC patients were significantly increased compared with those from healthy controls (5.7% ± 1.4% vs 3.4% ± 1.2%, *p* = 0.001), which was consistent with previous reports [24,25]. Another important cytokine, IFN-γ, showed a similar tendency as IL-21. However, there were no significant differences in the Tfh cell secretion of other cytokines (e.g., IL-17, IL-10) among those three groups (Fig. 4).

**Fig 5:**
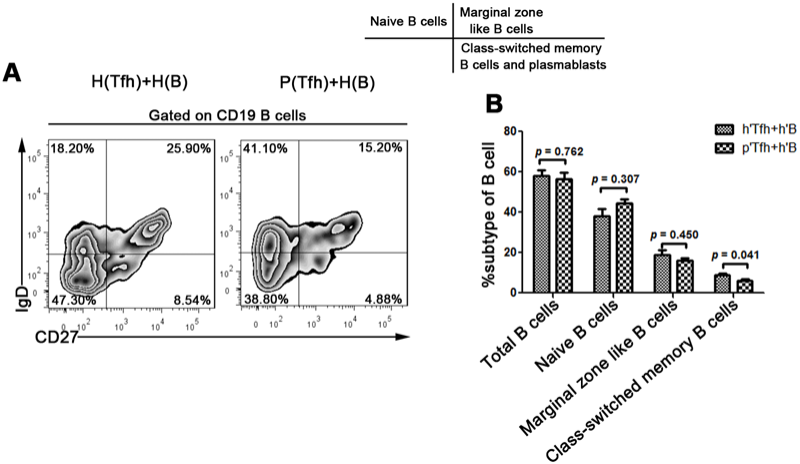
Tfh cells from HCC patients suppress the maturation of autologous B cells. (A) Circulating CXCR5+CD4+ Tfh cells from HCC patients (pTfh) or healthy controls (hTfh) and allogeneic CD19+ B cells from healthy controls (hB) are incubated at a ratio of 1:1 in the presence of SEB (100 ng/ml) in RPMI1640 complete medium; after 7–8 days of culture, the B cell subpopulations are examined by the surface phenotype. The data are a representative HCC patient and healthy control. (B) Statistical analysis showing that class-switched memory B cells are significantly decreased in the co-culture systems of pTfh and allogeneic hB compared with hTfh and hB. The data are from 10 HCC individuals and 10 healthy controls.

In addition, we further investigated the influence of Tfh cells on B cells in HCC patients. Circulating Tfh cells from 10 HCC (S1 Table) and 10 HC were sorted and co-cultured with allogeneic B cells from healthy donors in 1:1 as the suitable ratio (S2 Fig.). To mimic the antigen-specific interaction between T and B cells, SEB was added to the culture. B cells can be divided into IgD+CD27- naïve B cells, IgD+CD27+ marginal zone-like B cells, IgD-CD27+ class-switched memory B cells and plasmablasts [26]. As shown in Fig. 5, the frequency of class-switched memory B cells and plasmablasts was significantly decreased in the HCC patient co-culture systems compared with those from the healthy controls (hB) (6.3% ± 2.2% vs 8.8% ± 2.7%, *p* = 0.041). All the above data indicated that Tfh cell function in HCC patients was impaired and might disturb the ability of humoral immunity to prevent tumorigenesis.

### Decreased frequency of circulating CXCR5+CD4+ Tfh predicts poor survival in HCC patients

**Fig 6:**
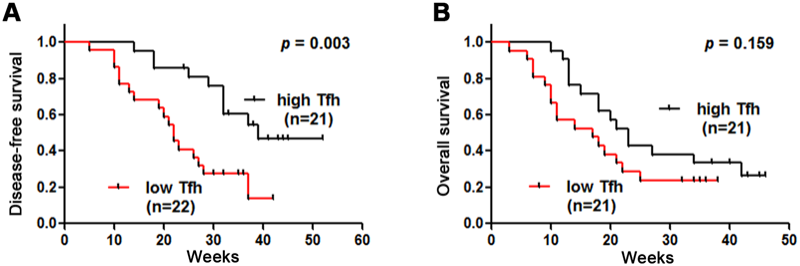
Decreased prevalence of circulating CXCR5+CD4+ Tfh cells predicts a poor survival in HCC. (A) 43 HCC patients in early stages of the disease (BCLC 0, A, B) are divided into two groups according to the median value of peripheral Tfh cells frequency (median value for BCLC 0, A and B, 9.4%). The low-Tfh group (n = 22, Tfh frequency: 6.7% ± 1.8%) has significantly poorer disease-free survival (DFS) rates compared to the high-Tfh group (n = 21, Tfh frequency: 11.8% ± 1.8%) (*p* = 0.003). (B) The other 42 HCC patients with advanced disease (BCLC C, D) are also divided into two groups according to the median value of peripheral Tfh cells frequency (median value for BCLC C and D, 7.4%). However, the circulating Tfh cells don’t have any significant predictive value (5.4% ± 1.5% vs 10.1% ± 3.1%, *p* = 0.159) for overall survival (OS) rates. The DFS and OS rates are analyzed by the Kaplan-Meier method, and the long-rank test is applied between-group comparisons.

To further address whether a decreased frequency of circulating Tfh cells is associated with HCC progression, 43 HCC patients with early-stage disease (BCLC 0, A, B) were divided into two groups according to the median value of peripheral Tfh cells frequency (median value for BCLC 0, A and B, 9.4%). The results showed that the low-Tfh group (n = 22, Tfh frequency: 6.7% ± 1.8%) had significantly poorer disease-free survival (DFS) rates compared with the high-Tfh group (n = 21, Tfh frequency: 11.8% ± 1.8%) (*p* = 0.003) (Fig. 6A). To investigate the overall survival (OS) rates, the other 42 HCC patients at an advanced stage (BCLC C, D) were also divided into two groups according to the median value of peripheral Tfh cells frequency (median value for BCLC C and D, 7.4%). However, circulating Tfh cells did not exhibit any significant predictive value (5.4% ± 1.5% vs 10.1% ± 3.1%, *p* = 0.159) for overall survival (OS) rates (Fig. 6B). The results suggested that the frequency of circulating Tfh cells might be considered as a predictor of poor survival in HCC patients.

## Discussion

CD4+ T cells represent a major component of the adaptive immune response and have been shown to be an integral part of the activation and regulation of host responses to many pathogens. Upon stimulation, naïve CD4+ T cells differentiate into effector cells known as T helper (Th) cells, and the distinct CD4+ T cell subsets have variable impact on tumor growth. Th1 cells and Th2 cells exhibit beneficial antitumor effects. Treg cells act to dampen antitumor immunity by suppressing the effector functions of a variety of immune cells, including Th1 cells, CD8+ T cells, NK cells, and tumor-infiltrating DCs. In contrast, the role of Th17 cells in tumorigenesis is still under debate [27–29]. Our previous study found that Treg cells [30] and CD4+ cytotoxic T cells [31] are involved in the development of HCC. However, limited information is available on the functional roles of Tfh cells in human cancers. The present study comprehensively characterized Tfh cells from HCC patients and found that a reduced frequency of Tfh cells is associated with a high rate of HCC recurrence.

Although the definition of Tfh cells is mainly dependent on the anatomical location, accumulating evidence has indicated the existence of circulating counterparts. Such circulating CXCR5+CD4+ T cells share phenotypic and functional similarities with Tfh cells from human secondary lymphoid organs [32,33]. Many studies have shown that an increased number of circulating CXCR5+CD4+ Tfh cells is associated with several of inflammatory diseases, such as CHB [25] and autoimmune diseases (systemic lupus erythematosus [34,35], ankylosing spondylitis [36,37], rheumatoid arthritis [38,39], polyangiitis [40], myasthenia gravis [41], and Sjögren’s syndrome [42]). In our study, we first found that a decrease in and functional impairment of circulating CXCR5+CD4+ Tfh cells are related to the development of HCC. It has been reported in breast cancer [15] and colorectal cancer [16] that liver-infiltrated Tfh cells also largely accumulate in nontumor regions as opposed to tumor regions. With regard to our data, more peripheral CXCR5+CD4+ Tfh cells might be recruited to liver tissues in response to the tumor, ultimately leading to a decrease in circulating CXCR5+CD4+ Tfh cells in HCC patients. Nonetheless, the relative mechanisms require further study.

IL-21 is mainly secreted by Tfh cells and plays a key role in their functions. The effects of IL-21 are pleiotropic due to the broad cellular distribution of the IL-21 receptor, and this cytokine plays a critical role in T cell-dependent and-independent human B cell differentiation in the generation of humoral immune responses [23,43]. We found that the peripheral Tfh cells from HCC patients exhibit a functional deficiency in producing IL-21 compared with those from HBV-LC and HC patients. The generation of high-affinity antibodies from antigen-specific memory B cells and plasma cells has a critical role in the neutralization and clearance of pathogens. Although the roles of B cells in cancer immunity are currently unclear, they could potentially affect antitumor immune responses via their antibody-producing capacities, APC functions, and/or production of specific cytokines [44,45]. In the B cell maturation assay, we found that Tfh cells from HCC patients showed decreased capacity to promote B cell maturation in comparison to cells from healthy controls (*p* = 0.041). This finding indicated that the maturation processes of B cells were disturbed by Tfh cells from HCC patients, and thus the various functions of B cells were suppressed in the HCC patients. All these data suggest that the functional impairment of Tfh cells might hinder antitumor immune responses in HCC patients.

As previously reported, the level of serum α-fetoprotein (AFP) is increased in most HCC patients [46]. Therefore, the measurement of serum AFP is important in the diagnosis of HCC and for monitoring response to various treatment modalities [47]. In our study, we found an increased serum level of AFP along with the notably decreased frequency of circulating Tfh cells in HCC patients (Fig. 3). It is well known that one of the biological properties of AFP is its regulatory effects on the immune response [48]. Shahriar Behboudi found that high concentrations of AFP suppress immune cell function in vitro [48] and that the function of CD4+ T cells isolated from HCC patients with high concentrations of serum AFP was impaired [49]. Further studies revealed that CD4+ T cell response is expanded in the early stages of HCC, usually associated with low concentrations of serum AFP, whereas this response was exhausted in later stages because of the high concentration of serum AFP [50]. Considering the immune suppression function of serum AFP, the decreased frequency or impaired function of Tfh cells in HCC might partially result from the effect of serum AFP.

Recently, a decreased number of infiltrating Tfh cells was reported to be a predictive survival marker for tumor patients. Chunyan Gu-Trantien [15] evaluated the association of Tfh cells with disease-free survival (DFS) in systemically untreated primary breast cancer (BC) and found that Tfh cells were a consistently prognostic marker and had a linear association with survival in the entire BC population as well as in the 2 main BC subtypes (HER2+ and ER+/HER2-). To validate the importance of Tfh-cell-related major soluble factors, Gabriela Bindea [16] investigated the expression of CXCL13, CXCR5, IL-21, and B-cell-related markers (MS4A1 and CD19) in relation to DFS in colorectal cancer; high Tfh cell marker (CXCL13 and CXCR5) expression was correlated with a significantly prolonged DFS. In the present study, our data showed that the low percentages of peripheral Tfh cells was associated with significantly poorer DFS rates compared to high percentages of Tfh cells (*p* = 0.003, Fig. 6A). Therefore, circulating Tfh cells may serve as a potential prognostic marker for HCC recurrence.

There are some limitations in our study. First, although the HCC patients enrolled in our study covered all stages from 0 to D based on BCLC criteria, the number of patients in stage 0 and stage D were few, which may have affected the results. Second, based on immunohistochemical staining, we did find Tfh cells in tumor regions, but their relationship with the progression of HCC and survival rate was not clear. To further resolve this question, additional tumor tissues with different grading and staging are being recruiting by our group. Third, we suggested that increased serum AFP might partially suppress the frequency and function of circulating Tfh cells, but experimental data to support this are lacking. Additionally, the detailed molecular mechanisms affecting the frequency and function of Tfh cells in HCC require further study.

In conclusion, our findings demonstrate that a progressive decrease in the number of Tfh cells is closely associated with HCC progression and poor survival rates in HCC patients. The direct and indirect effects of decreases in Tfh cells may be involved in the impairment of immune responses in HCC patients. These data highlight the novel role of Tfh cells in the immunocompromised status of HCC patients and also provide a potential therapeutic target for the treatment of HCC.

## Supporting Information

S1 Fig Surface markers profile of Tfh cells. (A) Representative dot plots of CD40L and BTLA staining on CXCR5+CD4+ Tfh cells from individual subjects in HCC patients and healthy controls are shown, statistical analysis showing that there are no significant differences regarding the frequency of CD40L+CXCR5+CD4+ Tfh cells and BTLA+CXCR5+CD4+ Tfh cells between HCC and healthy controls. Each dot represents one individual. *p* values are shown. (B) Representative dot plots depict the expression of the activation markers CD38, CD69, CD25 and HLA-DR on CXCR5+CD4+ Tfh cells from HCC patients and healthy controls, pooled data show that CD38+CXCR5+CD4+ Tfh cells are significantly decreased in HCC patients. Each dot represents one individual. *p* values are shown. (C) Representative dot plots depict the percentage of T cell subsets in HCC patients and healthy controls. T cell subsets are defined as: naïve T cells (CD45RA+CCR7+), central memory T cells (CD45RA-CCR7+), effector memory T cells (CD45RA-CCR7-), effector T cells (CD45RA+CCR7-). There are no significant differences regarding percentage of memory Tfh cells versus naïve Tfh cells between HCC and healthy controls. *p* values are shown.
(TIF)

S2 Fig Tfh cells co-culture with B cells at various ratios. Circulating Tfh cells from HCC patients are cultured with CD19+ B cells from HC at a ratio of 1:1, 1:5, 1:10 on SEB stimulation for 7 days. Statistical analysis showing that there are no significant differences about class-switched memory B cells between ratios. The data are from 4 HCC individuals and 4 healthy controls.
(TIF)

S1 Table Clinical characteristics of the 10 HBV-related HCC patients. ALT, alanine aminotransferase; AST, aspartate aminotransferase; AFP, α-fetoprotein; BCLC, Barcelona Clinic Liver Cancer; ND, no data.
(DOCX)

S1 Text Supporting text. This file contains detailed methods, including measurement of circulating Tfh cells’ other surface markers (CD40L, BTLA), markers of activation (CD38, CD69, CD25, HLA-DR), and percentage of memory Tfh cells versus naïve Tfh cells in the peripheral blood by flow cytometry, and co-culture of Tfh cells and B cells at various ratios.
(DOCX)
