# Supplementary figures and images for: Impaired Function of CD4+ T Follicular Helper (Tfh) Cells Associated with Hepatocellular Carcinoma Progression
Source: PLoS One. 2015 Feb 17;10(2):e0117458. doi: 10.1371/journal.pone.0117458 (PMC4331507; doi:10.1371/journal.pone.0117458)

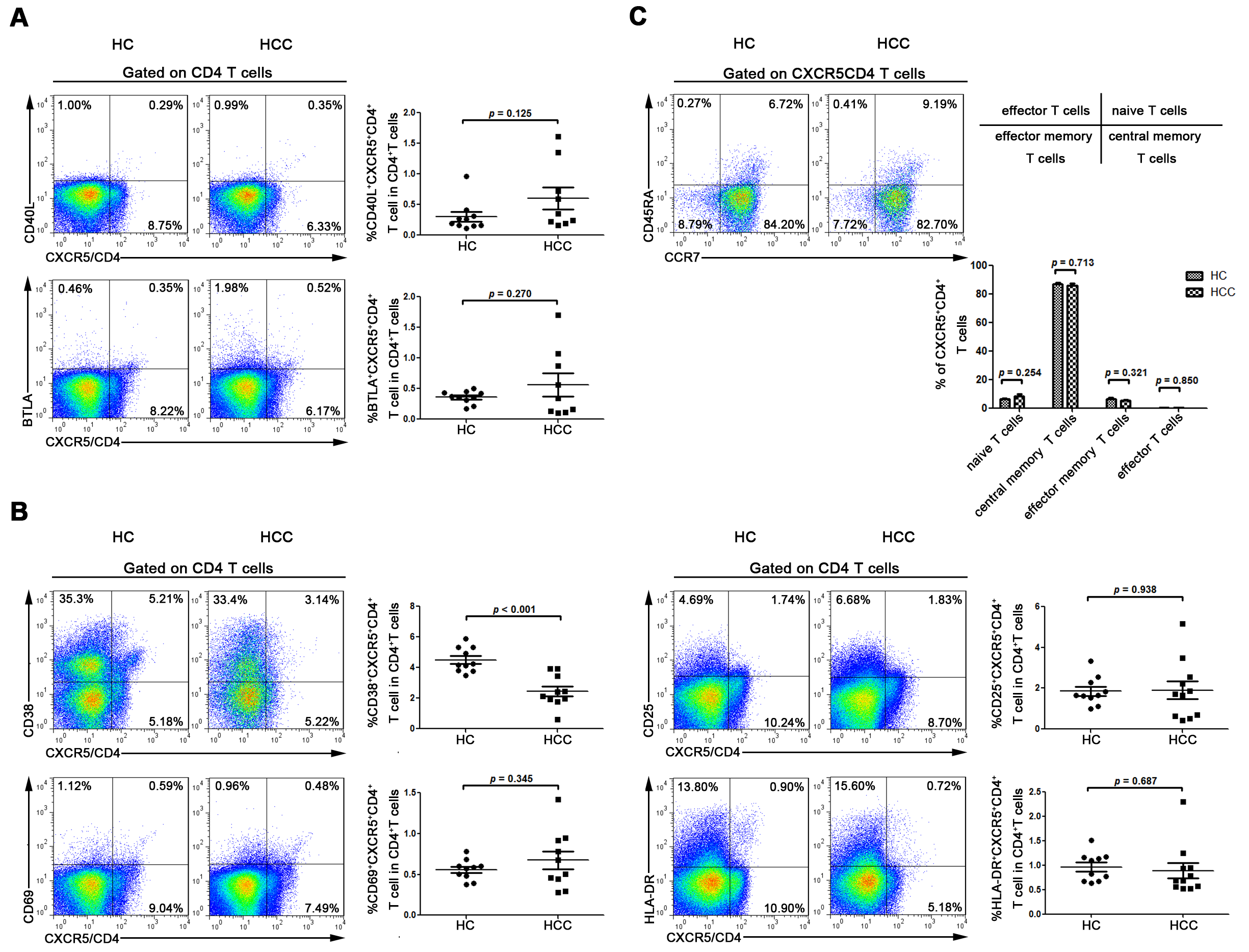

Supplement: S1 Fig — (A) Representative dot plots of CD40L and BTLA staining on CXCR5+CD4+ Tfh cells from individual subjects in HCC patients and healthy controls are shown, statistical analysis showing that there are no significant differences regarding the frequency of CD40L+CXCR5+CD4+ Tfh cells and BTLA+CXCR5+CD4+ Tfh cells between HCC and healthy controls. Each dot represents one individual. p values are shown. (B) Representative dot plots depict the expression of the activation markers CD38, CD69, CD25 and HLA-DR on CXCR5+CD4+ Tfh cells from HCC patients and healthy controls, pooled data show that CD38+CXCR5+CD4+ Tfh cells are significantly decreased in HCC patients. Each dot represents one individual. p values are shown. (C) Representative dot plots depict the percentage of T cell subsets in HCC patients and healthy controls. T cell subsets are defined as: naïve T cells (CD45RA+CCR7+), central memory T cells (CD45RA-CCR7+), effector memory T cells (CD45RA-CCR7-), effector T cells (CD45RA+CCR7-). There are no significant differences regarding percentage of memory Tfh cells versus naïve Tfh cells between HCC and healthy controls. p values are shown. (TIF) [file pone.0117458.s001.tif]

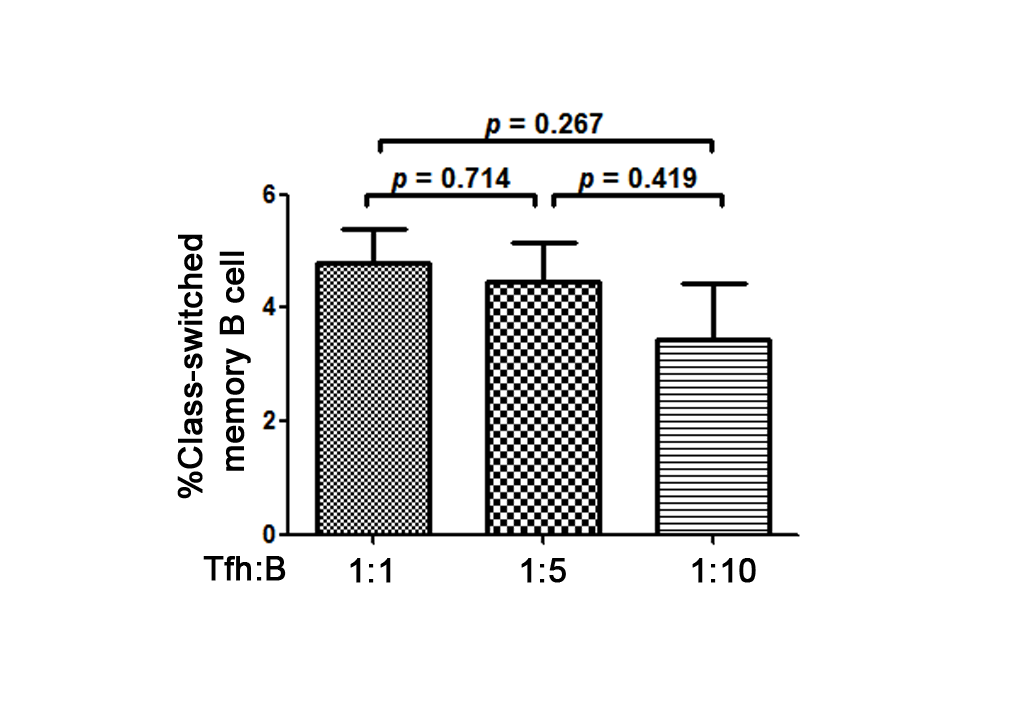

Supplement: S2 Fig — Circulating Tfh cells from HCC patients are cultured with CD19+ B cells from HC at a ratio of 1:1, 1:5, 1:10 on SEB stimulation for 7 days. Statistical analysis showing that there are no significant differences about class-switched memory B cells between ratios. The data are from 4 HCC individuals and 4 healthy controls. (TIF) [file pone.0117458.s002.tif]
