# Supplementary material for: Impaired Function of CD4+ T Follicular Helper (Tfh) Cells Associated with Hepatocellular Carcinoma Progression
Source: PLoS One. 2015 Feb 17;10(2):e0117458. doi: 10.1371/journal.pone.0117458 (PMC4331507; doi:10.1371/journal.pone.0117458)
Supplement: S1 Table — ALT, alanine aminotransferase; AST, aspartate aminotransferase; AFP, α-fetoprotein; BCLC, Barcelona Clinic Liver Cancer; ND, no data. (DOCX) [file pone.0117458.s003.docx]

**Table S1. Clinical characteristics of the 10 HBV-related HCC patients**

|  | **ALT (U/L)** | **AST (U/L)** | **HBV DNA (IU/ml)** | **AFP (ng/ml)** | **BCLC** | **Child-Pugh** |
| --- | --- | --- | --- | --- | --- | --- |
| **Patient-1** | 16 | 84 | 4.43 × 10^3^ | 3607 | C | B |
| **Patient-2** | 20 | 39 | ND | 6 | A | A |
| **Patient-3** | 57 | 45 | 7.27 × 10^5^ | 1585 | C | A |
| **Patient-4** | 366 | 428 | ND | 1248 | B | A |
| **Patient-5** | 92 | 87 | 1.83 × 10^6^ | 3985 | C | A |
| **Patient-6** | 82 | 74 | 3.64 × 10^4^ | ˃ 20000 | C | B |
| **Patient-7** | 38 | 46 | ND | 1883 | B | B |
| **Patient-8** | 32 | 37 | 1.06 × 10^3^ | ˃ 20000 | A | A |
| **Patient-9** | 31 | 61 | 6.38 × 10^3^ | 35 | A | B |
| **Patient-10** | 25 | 43 | 5.88 × 10^8^ | 28 | B | B |

ALT, alanine aminotransferase; AST, aspartate aminotransferase; AFP, α-fetoprotein; BCLC, Barcelona Clinic Liver Cancer; ND, no data.
