# Supplementary material for: Impaired Function of CD4+ T Follicular Helper (Tfh) Cells Associated with Hepatocellular Carcinoma Progression
Source: PLoS One. 2015 Feb 17;10(2):e0117458. doi: 10.1371/journal.pone.0117458 (PMC4331507; doi:10.1371/journal.pone.0117458)
Supplement: S1 Text — This file contains detailed methods, including measurement of circulating Tfh cells’ other surface markers (CD40L, BTLA), markers of activation (CD38, CD69, CD25, HLA-DR), and percentage of memory Tfh cells versus naïve Tfh cells in the peripheral blood by flow cytometry, and co-culture of Tfh cells and B cells at various ratios. (DOCX) [file pone.0117458.s004.docx]

**Supplemental methods**

Flow cytometric analysis

PE-conjugated anti-CD4, APC-conjugated anti-CD4, Alexa Fluor 488-conjugated anti-CXCR5, APC-conjugated anti-CD45RA and PE-conjugated anti-BTLA were purchased from BD PharMingen (San Diego, CA, USA). PerCP-conjugated anti-CCR7, PE-conjugated anti-CD69 and APC-conjugated anti-CD40L were purchased from Biolegend (San Diego, CA, USA). PE-conjugated anti-CD25, PerCP-conjugated anti-HLA-DR, PerCP-conjugated anti-CD4 and APC-conjugated anti-CD38 were purchased from BD Biosciences (San Diego, CA, USA).

To determine circulating Tfh cells’ other surface markers (CD40L, BTLA), markers of activation (CD38, CD69, CD25, HLA-DR), and percentage of memory Tfh cells versus naïve Tfh cells in the peripheral blood, PBMCs (at least 1×10^6^ cells/tube) were stained with mAbs for 30 min on ice. Appropriate isotype antibody controls were used for each sample. The cells were washed and examined by four-color flow cytometry.

Co-culture of Tfh cells and B cells

Circulating CXCR5+CD4+ Tfh cells from 4 HCC patients (pTfh) or 4 healthy controls (hTfh) and allogeneic CD19+ B cells from 4 healthy controls (hB) were sorted using a FACSAria II cell sorter and incubated with each other at ratios of 1:1, 1:5, 1:10 in the present of SEB (100 ng/ml) in RPMI1640 complete medium supplemented with 2 mM glutamine, 1%(v) nonessential amino acids, 1% sodium pyruvate, penicillin (50 U/ml), streptomycin (50 µg/ml), kanamycin (50 µg/ml), and 10%(v) fetal bovine serum (all from Hyclone). After 7-8 days of culture, B-cell subpopulations were examined by surface phenotypes.
